# Chicken adaptive response to nutrient density: immune function change revealed by transcriptomic analysis of spleen

**DOI:** 10.3389/fimmu.2023.1188940

**Published:** 2023-05-15

**Authors:** Yan Zhou, Dingguo Cao, Jie Liu, Fuwei Li, Haixia Han, Qiuxia Lei, Wei Liu, Dapeng Li, Jie Wang

**Affiliations:** Poultry Breeding Engineering Technology Center of Shandong, Poultry Institute, Shandong Academy of Agricultural Sciences, Jinan, Shandong, China

**Keywords:** nutrient density, metabolizable energy, crude protein, spleen, WGCNA

## Abstract

Feed accounts for the largest portion (65-70%) of poultry production costs. The feed formulation is generally improved to efficiently meet the nutritional needs of chickens by reducing the proportion of crude protein (CP) and metabolizable energy (ME) levels in the diet. Although many studies have investigated the production performance during dietary restriction, there is a lack of research on the mechanisms by which immune cell function is altered. This study examined the effects of ME and CP restriction in the chicken diet on serum immunoglobulins and expression of immune function genes in spleen. Changes in serum immunoglobulins and immune-related gene expression were analyzed in 216 YS-909 broilers fed with 9 different dietary treatments, including experimental treatment diets containing low, standard, and high levels of ME or CP in the diet. At 42 days of age, serum immunoglobulins and expression of spleen immune genes in 6 female chickens selected randomly from each dietary treatment (3×3 factorial arrangement) group were measured by enzyme-linked immunosorbent assay (ELISA) and transcriptomic analysis using RNA sequencing, respectively. The results showed that the IgM level in the low ME group chickens was significantly (*p* < 0.05) lower than that in other groups. In addition, immune-related genes, such as *MX1, USP18, TLR4, IFNG* and *IL18* were significantly upregulated when the dietary nutrient density was reduced, which may put the body in an inflammatory state. This study provided general information on the molecular mechanism of the spleen immune response to variable nutrient density.

## 1 Introduction

As investing in the immune system is expensive, adequate nutrition is essential to health, and the availability of resources has long been recognized as a critical factor in the immune response (1). Since nearly every nutrient in the diet contributes to sustaining an “optimal” immune response, inadequate or excessive dietary intake can harm the immune system and increase susceptibility to a variety of pathogens (2). Diet has a profound impact on immunological functions, affecting both humoral and cellular immune functions. Additionally, the interaction of the immune system with pathogens and other systems in the body can also be profoundly affected by dietary restrictions or excesses (3). The immune and metabolic systems must mutually interact for the host to maintain homeostasis (4). Dietary intake, energy utilization and storage are connected to immune regulation of tissue function by integrated immunometabolic responses and such connection is essential for the maintenance and restoration of homeostasis (4–6). Energy imbalance results in impaired immune and metabolic homeostasis, leading to the infiltration of inflammatory immune cells, such as TNF- and IL-6-producing lipid-associated macrophages (LAMs), IFNG-producing CD4^+^ T cells, neutrophils, IgG2C-producing B cells, and cytotoxic CD8^+^ T cells (4). Dietary protein and amino acids are important for the proper functioning of the immune system (7). Many early studies on immune defense mechanisms in the context of protein malnutrition have revealed the changes in both macrophage and lymphocyte responses to infection. One of the pathways activated during the restriction of proteins and amino acids is the autophagy pathway mediated by the integrated stress response, transsulfurization pathway and complex stress response to regulate the immune response, which is another way to solve the problem of protein restriction (PR) and amino acid restriction (8–10).

Although a variety of metabolic adaptations that result from dietary restriction have been the subject of numerous studies, little is known about how immune cell function is affected. We hypothesized that different diet combinations of metabolizable energy (ME) and crude Protein (CP) might have an impact on the body’s immune function by influencing gene expression in the spleen. Therefore, we designed the experiments in this study to test our hypothesis, as follows: 216 chicks were weighed individually and randomly allocated to test cages, and subsequently randomly assigned to experimental diets with different ME/kg or CP. At 42 days of age, 2 female chickens from each treatment diet (3×3 factorial arrangement, 3 times) were analyzed for serum immunoglobulins and spleen immune-related gene expression. The goal of this study is to elucidate the mechanisms by which immune cell function is altered in spleen during dietary restriction.

## 2 Methods

### 2.1 Ethics statement

This study was conducted in accordance with the guidelines of the Ministry of Science and Technology of China. All the procedures were approved by the scientific committee of the Shandong Academy of Agricultural Sciences (Jinan, China) (SAAS-2022-a35).

### 2.2 Animals and sample collection

A total of 216 YS-909 chicks obtained from the Poultry Institute, Shandong Academy of Agricultural Sciences (Jinan, China) were used in this study. Among them, 54 female chickens were randomly selected for measuring serum immunoglobulin levels by enzyme-linked immunosorbent assay (ELISA) and 30 female chicks for gene expression analysis by RNA-sequencing. All chickens were raised under standard brooding and rearing conditions. At 0 days of age, 216 chicks were weighed individually and randomly assigned to test cages (30×45×45 cm) in a test house. The above chickens were divided into 9 groups with 24 chickens per group, 3 replicates per group and 8 chickens per replicate. In a 3×3 factorial arrangement, the pullets were randomly assigned to experimental diets with 2,850, 2,950 and 3,050 kcal of ME/kg of diet each containing 20, 21, and 22% of CP (**Table 1**; **Tables S1**, **S2**). The pullets were fed diets in powder form and provided ad libitum access to feed and water. Each dietary treatment was replicated 3 times. At 42 days of age, 2 female chickens from each dietary treatment (3×3 factorial arrangement, 3 times) were randomly selected for blood and spleen tissue collection. The spleen was frozen immediately and stored at -80°C until assayed.

**Table 1:** Composition of experimental diets and descriptive statistics of natural antibody titers.

Group	CP	ME	ME (kcal/kg)	CP (%)	IgM(ng/ml)	IgG(ug/ml)	IgA(ug/ml)
			1-21d/22-42d	1-21d/22-42d			
Group1	Low	Low	2850/2950	20/18	221.94^C^	11.86	30.91^B^
Group2	Normal	Low	2850/2950	21/19	290.81^BC^	13.63	34.79^B^
Group3	High	Low	2850/2950	22/20	283.8^BC^	13.22	33.74^B^
Group4	Low	Normal	2950/3050	20/18	268.14^BC^	12.40	31.76^B^
Group5	Normal	Normal	2950/3050	21/19	308.61^BC^	13.68	35.19^B^
Group6	High	Normal	2950/3050	22/20	449.85^A^	11.62	33.30^B^
Group7	Low	High	3050/3150	20/18	373.78^AB^	11.38	43.52^A^
Group8	Normal	High	3050/3150	21/19	309.68^CB^	12.42	36.78^AB^
Group9	High	High	3050/3150	22/20	307.39^BC^	11.69	29.27^B^
	SEM				91.19	4.68	5.955
CP	Low	–			287.95	11.88	35.397
	Normal	–			303.03	12.24	35.585
	High	–			347.02	12.18	32.106
ME	–	Low			265.52^B^	12.90	33.147
	–	Normal			342.2^A^	12.57	33.417
	–	High			330.28^A^	11.83	36.524
*p*-value	CP				NS	NS	NS
	ME				0.03	NS	NS
	CP*ME				0.02	NS	0.01

*ME, metabolizable energy; CP, crude protein.

The majuscule letters in the shoulder label indicate significant differences (P < 0.05).

"NS" indicates no significant difference (P > 0.05).

### 2.3 Enzyme-linked immunosorbent assay

Chicken immunoglobulin M (IgM), immunoglobulin G (IgG) and immunoglobulin A (IgA) levels in the serum were measured using a chicken-specific ELISA kit (Wuhan ColorfuiGene Biological Technology Co., Ltd., Wuhan, China) with a sandwich ELISA. The microelisa strip plate provided in the kit was pre-coated with an antibody specific to IgM/IgA/IgG. The optical density (OD) was measured using a spectrophotometer at a wavelength of 450 nm. The concentration of IgM/IgA/IgG in the samples was determined by comparing the OD of the samples to the standard curve. Blood samples were homogenized by whirlpool oscillation at room temperature and centrifuged (1,000 g, 20 min) to separate the debris and the pellet. The supernatant was frozen immediately and stored at -80°C until assayed. The assay was performed in accordance with the manufacturer’s protocol and suggested dilutions to optimize accuracy.

### 2.4 RNA sequencing and data analysis

A genome-wide dataset by RNA sequencing to determine the gene expression profile in spleen tissue in the different groups (Group 2, 4, 5, 6, 8) (n=6, total 30 birds). The raw data of the genome-sequence were deposited in the BIG Data Center repository (Beijing Institute of Genomics (BIG), Chinese Academy of Sciences, Beijing, China). The database is publicly accessible at: https://bigd.ac.cn/gsa (PRJCA015332, CRA010198). Gene expression profile analysis by ultra-high-throughput sequencing on the Illumina Novaseq 6000 Platform (Illumina Inc., San Diego, CA, USA) was performed by Berry Genomics (Beijing, China) (**Table S3**). The bcl2fastq (Illumina Inc.) was used to convert raw data to FASTQ files. Clean reads were obtained by removing reads with low-quality and adapter sequences and mapped to the reference chicken genome and genes (GRCg6a, Ensembl release 106) using TopHat 1.3.2 (https://ccb.jhu.edu/software/tophat). Gene expression levels were calculated using the Reads Per Kilobase of transcript per Million (RPKM) method (11). The DEseq2 software was used to analyze the significance of differences in gene expression levels for differential expression analysis without biological duplication (12). The standard for screening the total differentially expressed genes (DEGs) is log2FC <- 1, log2FC>1, *p*<0.05.

### 2.5 Weighted gene co-expression network analysis

Using the data generated by RNA-sequencing analysis of the 30 individuals from the different groups of YS-909 chickens, the gene sets associated with phenotypes (ME, CP, IgA, IgM and IgG) were respectively identified by the weighted gene co-expression network analysis (WGCNA). Genes with the top 75% variance are used as input data according algorithm filters. The WGCNA was performed using the “WGCNA” package in the R software with the construction of an adjacency matrix and a topological overlap matrix (TOM) and calculating the corresponding dissimilarity (1-TOM) (the soft power values of ME chicken population = 12 and CP chicken population = 3) (13). The dynamic tree cutting method was used to construct the gene dendrogram map and identify the module. Then, the correlation between the characteristic genes of the module and the survival conditions was calculated (14). Further analysis of the modules containing the target candidate genes was performed for differential gene expression analysis.

### 2.6 Comprehensive analysis of protein–protein interaction network

The Search Tool for the Retrieval of Interacting Genes (STRING) database was used to evaluate protein–protein interaction (PPI) data and gene ontology (GO) enrichment analysis (15). The network was clustered to a specified “Markov Clustering (MCL) inflation parameter”, and the inflation parameter was 3.

### 2.7 Statistical analyses

The significance of the differences between groups was tested by the 3×3 factorial arrangement using the SPSS software version 22.0 (IBM Corp., Armonk, NY, USA). Confidence limits were set at 95% and p < 0.05 was considered significant. Data are expressed as the mean ± standard error of the mean (SEM).

## 3 Results

### 3.1 Comparison of Ig levels in the different chicken groups

The assessment of the immune function in chickens from the ME and CP dietary treatment groups by measuring the serum levels of IgA, IgG and IgM revealed significant differences in the IgM levels between chickens from the low, high ME and normal control groups, as shown in **Table 1**. The IgM in the low ME chicken group of chickens was significantly (*p* < 0.05) lower than that in the normal and high ME chicken groups. There were no differences in the serum levels of IgG and IgA among chicken groups 1-9.

### 3.2 Construction of the WGCNA and module identification

#### 3.2.1 ME data set

**Figure 1:**
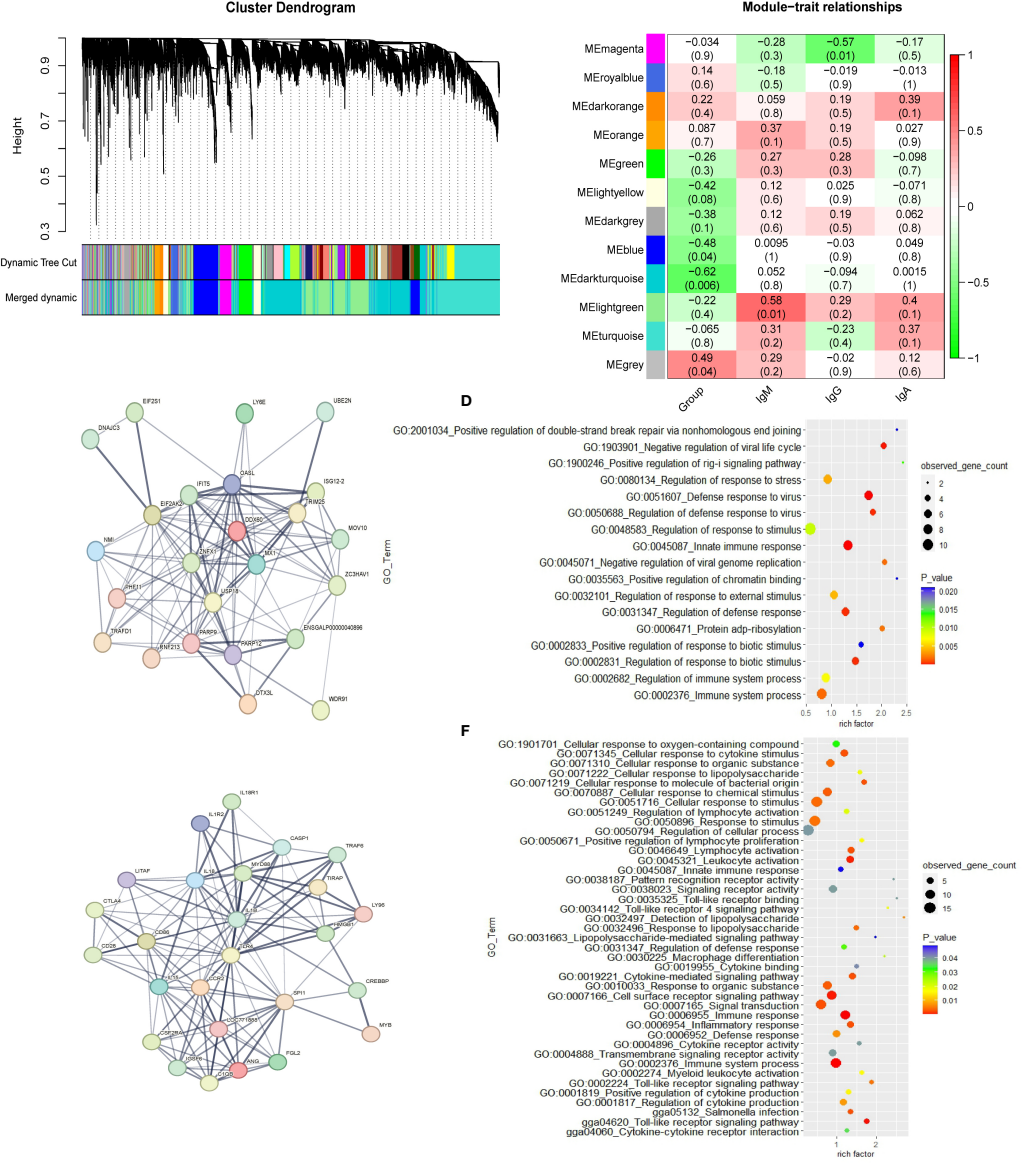
The WGCNA results from the ME data set. **(A)** Module clustering tree diagram. **(B)** Relationship between gene modules in chicken spleen tissue and ME traits. Each row in the Table corresponds to a module, and each column corresponds to a trait. Each cell contains the corresponding correlation and *p*-value. The module name is shown on the left side of the heatmap. The correlation is shown on the right side of the heat map, according to the color legend (positive correlation in red, negative correlation in green). **(C)** Protein-protein interaction (PPI) network of cluster 1 in the blue module. Each node represents a protein, and the lines between the nodes indicate that they interact with the protein. **(D)** GO enrichment results of cluster 1 in the blue module. **(E)** Protein-protein interaction (PPI) network of cluster 2 in the blue module. **(F)** GO enrichment results of cluster 2 in the blue module.

The weighted gene co-expression network was constructed with 16,205 genes. Gene modules were obtained according to the differences in hierarchical clustering, as shown in **Figure 1A**. According to the height value corresponding to the gene co-expression similarity between modules, the modules whose value is less than 0.35 are merged. A total of twelve modules were identified after merging. These gene co-expression modules are all represented and assigned different colors (**Figure 1B**). The gene modules most related to the ME group were determined. The blue module (r = -0.48; *p* = 0.04), dark turquoise module (r=-0.62; *p*= 0.006) and grey module (r=0.49; *p*= 0.04) have significant correlation with the ME group in spleen tissue, which suggests that genes in these modules may play a key role in spleen function. The 1,542 genes in the blue module, which are highly related to the target traits, and the corresponding modules were clustered together. Some genes related to the classical immune function pathway, such as *MX1*, *DDX60*, *TLR4*, *MYD88* and *IL18* were also found in the blue module (**Table S4**). The network analysis (**Tables S5**, **S6**) of all the genes of the blue module in the YS 909 chicken WGCNA performed with the STRING software to predict the structure of the protein network revealed the existence of an antiviral regulatory network (**Figure 1C**) and an antibacterial regulatory network (**Figure 1E**). The GO enrichment analysis showed that the related genes were enriched to immune-related GO processes, such as GO term categories: GO:1903901 Negative regulation of viral life cycle, GO:0051607 Defense to virus, GO:0045087 Innate immune response and GO:0034142 Toll-like receptor 4 signaling pathway (**Figures 1D, F**).

#### 3.2.2 CP data set

**Figure 2:**
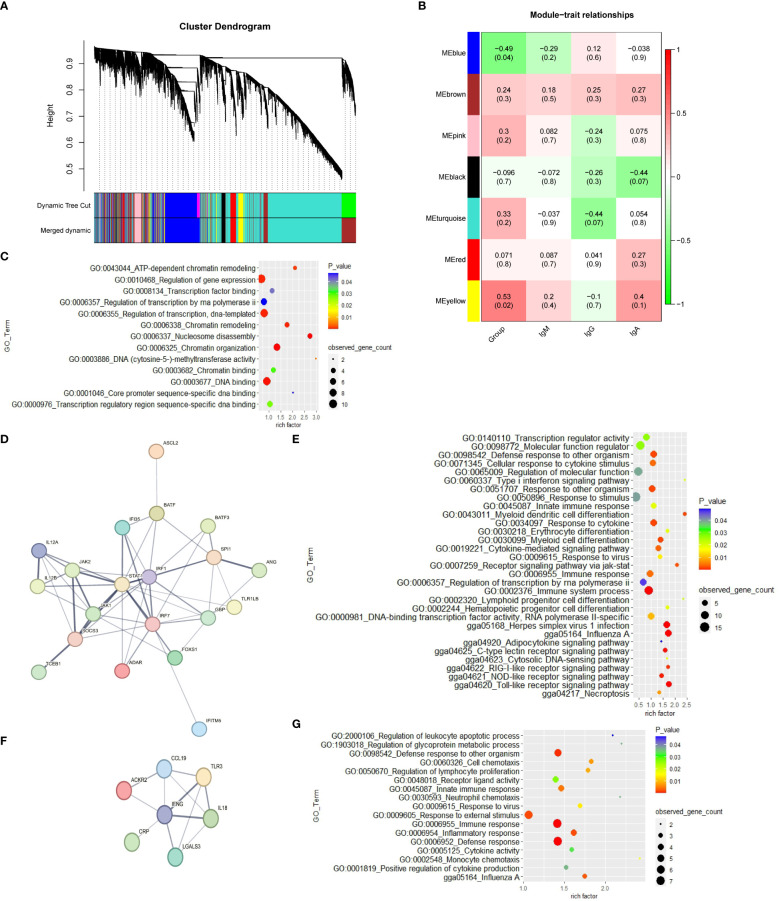
The WGCNA results from the CP data set. **(A)** Module clustering tree diagram. **(B)** Relationship between gene modules in chicken spleen tissue and ME traits. Each row in the Table corresponds to a module, and each column corresponds to a trait. Each cell contains the corresponding correlation and *p*-value. The module name is shown on the left side of the heatmap. The correlation is shown on the right side of the heat map, according to the color legend (positive correlation in red, negative correlation in green). **(C)** GO enrichment results of cluster 1 in the yellow module. **(D)** Protein-protein interaction (PPI) network of cluster 2 in the blue module. Each node represents a protein, and the lines between the nodes indicate that they interact with the protein. **(E)** GO enrichment results of cluster 2 in the blue module. **(F)** Protein-protein interaction (PPI) network of cluster 8 in the blue module. **(G)** GO enrichment results of cluster 8 in the blue module.

The weighted gene co-expression network was constructed with 16,097 genes. The gene modules were obtained based on the difference in hierarchical clustering (**Figure 2A**). A total of 7 modules were identified after merging. These co-expression modules are all represented in different colors in **Figure 2B**. The blue module (r = -0.49; *p* = 0.04) and yellow module (r=0.53; *p*= 0.02) have significant correlation with the CP group in spleen tissue, which suggests that genes in these modules may play a key role in spleen function. In the blue module, 3,117 genes that are highly correlated with the corresponding modules and traits were clustered together (**Table S7**). Some genes associated with the classical immune function pathway, such as *IRF1*, *IRF7*, *IFNG* and *IL18* were also found in the blue module. The data in **Table S9** show the correlation between the yellow module genes and CP traits. The GO results reveal that the yellow module genes are correlated with “nucleosome disassembly and regulation of gene expression” (**Table S9**; **Figure 2C**). The network analysis of all the genes of the blue module in the YS 909 chicken WGCNA performed with the STRING software to predict the structure of the protein network revealed is the existence of an immune system process network (**Figure 2D**) and a defense response network (**Figure 2F**; **Tables S10**, **S11**). GO enrichment analysis showed that the related genes were enriched to defense response -related GO processes, such as GO term categories: GO:0006955 Immune response, GO:0009615 response to virus and GO:0006952 defense response (**Figures 2E, G**).

### 3.3 Identification of significantly differentially expressed genes

**Figure 3:**
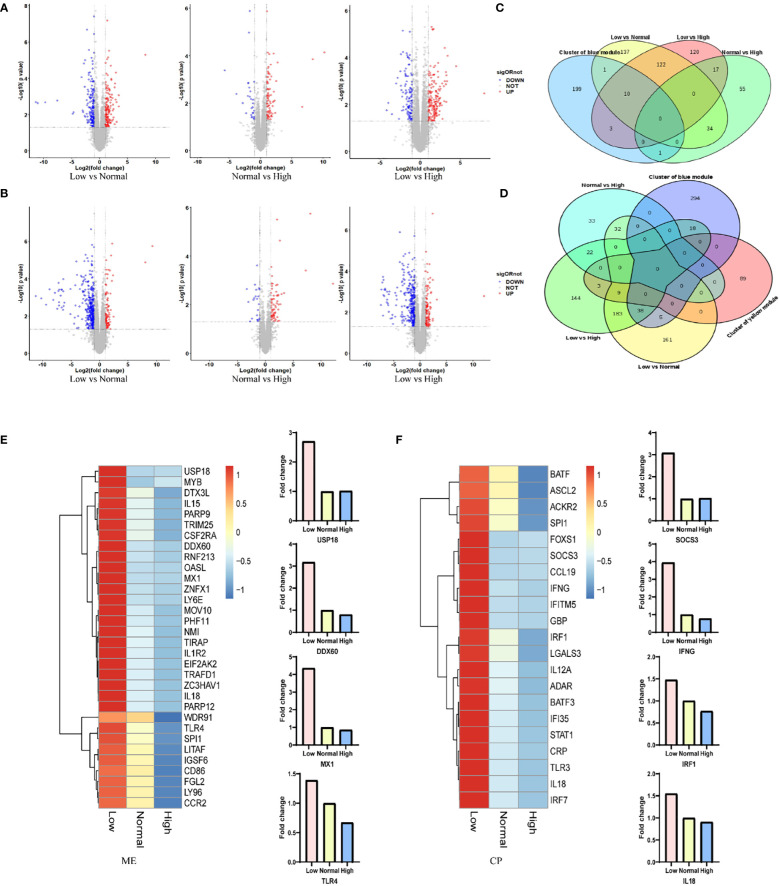
**(A, B)** Changes in gene expression profiles of chicken from the low, normal and high ME or CP groups. Red dots represent upregulated genes, blue dots represent downregulated genes, and grey dots (No) represent non-significantly DEGs. **(C, D)** The intersection results of network hub genes and DEGs for ME and CP treatments. **(E, F)** Expression levels (foldchange) of representative genes involved in the protein–protein interaction (PPI) network according to the transcriptome analysis data of chickens from the low, normal and high ME or CP groups.

RNA was extracted from the spleen of six representative 42-day old chickens per group. Comparison of the gene expression profile of the different ME or CP groups identified a total of 500 DEGs (|log2FC|≥1, with *p*<0.05) in spleen in the ME group (**Figure 3A**; **Table S12**) and 653 DEGs in the CP groups (**Figure 3B**; **Table S13**). In particular, we focused on the DEGs in the regulatory network formed by WGCNA. The intersection results of the network hub genes and DEGs are displayed in the form of a Venn plot (**Figures 3C, D**). As shown in **Figure 3E**, the expression levels of the *MX1*, *DDX60* and *USP18* genes in the antiviral-related regulatory network were negatively correlated with the ME level. When the ME level was reduced, the gene expression level in spleen was significantly upregulated. In addition, the *TLR4* gene, which is associated with the antibacterial pathway, also showed a similar pattern. Additionally, the *SOCS3*, *IFNG*, *IRF1* and *IL18* genes in the regulatory network associated with the immune response were significantly upregulated when dietary protein levels were decreased (**Figure 3F**).

## 4 Discussion

Consuming a diet that meets the energy demands of the body and provides essential nutrients contributes to a healthy immune system. Nutritional diseases due to malnutrition or overnutrition impair immune system function (16). Previous studies have shown that changing the amount of nutrients in the diet can alter the expression of immune-related genes, thereby regulating and reshaping immune responses. Considering that the spleen is one of the most important immune organs, and different dietary nutrient densities will lead to different probabilities of developing metabolic and infectious diseases, we expected to identify DEGs related to the spleen immune response elicited by different diets. The identification of these DEGs will lead to a better understanding of the immune mechanism of the spleen under specific conditions (17–20).

In the present study, we detected 1,153 DEGs (500 DEGs in ME groups and 653 DEGs in CP groups) between the spleen of CR-fed and PR-fed YS-909 chickens. Considering the results of the WGCNA, DEGs and Network analysis, the most statistically significant hub genes were upregulated. As a protein coding gene, the *MX1* (MX dynamin like GTPase 1) gene prevents the import of nuclear material into viral nucleocapsids, is suggested to have a disruptive effect on influenza A viruses (21). Diseases associated with *MX1* include influenza and viral encephalitis. Its related pathways are antiviral mechanism by interferon (IFN)-stimulated genes and cytokine signaling in the immune system. Other transcriptomic studies in poultry have also shown that the *MX1* gene is upregulated in the respiratory tract and ileum (22, 23). *DDX60* (DExD/H-box helicase 60), *TLR4* (toll like receptor 4) and *USP18* (ubiquitin specific peptidase 18) are all protein coding genes. Their related pathways are the “MyD88-dependent cascade” initiated on the endosome and the “overview of IFN-mediated signal pathway”. MyD88 serves as a central hub in innate immune responses, receiving signals from several receptors, located in the plasma membrane or in the endosome, that sense various types of pathogen-associated molecular patterns, located in the plasma membrane or in the endosome. Activation of the downstream NF-κb pathway *via* MyD88 produces IFNs and pro-inflammatory or anti-inflammatory cytokines to antagonize bacterial infections (24). IFN gamma (*IFNG*)-related pathways include gene expression (transcription) and interleukin-12 family signaling. GO annotations related to this gene include cytokine activity and type II IFN receptor binding. IFN Regulatory Factor 1(*IRF1*)-related pathways include “overview of IFN-mediated signal pathway”. Avian *IRF1* plays an important role in host innate immunity against viral infection (25). Interleukin 18 (*IL18*) binds to IL18RAP and IL18R1 to form a signaling ternary complex that activates the NF-κB signaling pathway and induces the synthesis of inflammatory mediators (26, 27). IL18 induces natural killer (NK) cells and T- helper cell 1 (Th1) cells to synthesize IFNG through synergistic action with interleukin 12 (IL12) (26, 27). IL18 synergizes with IL12/interleukin-12 to induce IFNG synthesis from T-helper 1 (Th1) cells and natural killer (NK) cells (28). In the future, it may be worthwhile to perform functional studies on these genes in spleen in a state of nutrient deficiency to better understand their function in the immune system.

Although our results provide general information on the molecular mechanisms and functional differences in the spleen under different nutrient density diets, this study has some limitations. For instance, the identification of the DEGs and the follow-up pathway/network analyses were conducted merely relying on a bioinformatics approach. Thus, extensive experimental validation work is still needed. Also, the interaction of gut microbes and nutritional metabolism through the liver-spleen axis with spleen immune function was not considered in the experimental design.

Collectively, our study identifies a set of negative feedback signals related to changes in nutrient density. Spleen genes associated with the immune response were significantly upregulated when dietary density levels were decreased. Although primarily descriptive, we provide evidence that nutrient density restriction can lead to upregulation of genes related to spleen immune function, which may put the body in an inflammatory state.

## Data availability statement

The datasets presented in this study can be found in online repositories. The names of the repository/repositories and accession number(s) can be found in the article/**Supplementary Material**.

## Ethics statement

The animal study was reviewed and approved by Science Research Department of the Shandong Academy of Agricultural Sciences (SAAS) (Ji’nan, China).

## Author contributions

YZ, JW and DC conceived and designed the study. YZ performed statistical analyses and drafted the paper. QL, YZ, HH, and WL participated in data analyses. JW, JL, FL, HH, and DL participated in the design of the study and contributed to data acquisition. All authors contributed to the article and approved the submitted version.
